# Polypharmacy in patients with epilepsy: A nationally representative cross-sectional study^☆^

**DOI:** 10.1016/j.yebeh.2020.107261

**Published:** 2020-07-03

**Authors:** Samuel W. Terman, Carole E. Aubert, Chloe E. Hill, Donovan T. Maust, John P. Betjemann, Cynthia M. Boyd, James F. Burke

**Affiliations:** a University of Michigan Department of Neurology, Ann Arbor, MI 48109, USA; b University of Michigan Institute for Healthcare Policy and Innovation, Ann Arbor, MI 48109, USA; c Department of General Internal Medicine, Bern University Hospital, University of Bern, Bern, Switzerland; d Institute of Primary Health Care (BIHAM), University of Bern, Bern, Switzerland; e Center for Clinical Management Research, VA Ann Arbor Healthcare System, Ann Arbor, MI 48109, USA; f University of Michigan Department of Psychiatry, Ann Arbor, MI 48109, USA; g University of California San Francisco, Weill Institute for Neurosciences, San Francisco, USA; h Johns Hopkins University, Center on Aging and Health, Baltimore, MD 21205, USA

**Keywords:** Epilepsy, Epidemiology, Opioids, Polypharmacy

## Abstract

**Objective:**

The objective of the study was to characterize the prevalence of polypharmacy and central nervous system (CNS)-acting medications in patients with epilepsy, and particular types of medications.

**Methods:**

This was a retrospective cross-sectional study using data from the nationally representative National Health and Nutrition Examination Survey (NHANES). We included patients who reported taking at least one prescription medication in order to treat seizures or epilepsy during NHANES survey years 2013–2016. We assessed the number and types of drugs and predictors of total number of medications using a negative binomial regression. We then assessed prevalence of polypharmacy (≥5 medications), CNS polypharmacy (≥3 CNS-acting medications) and additional CNS-acting medications, and drugs that lower the seizure threshold (i.e., bupropion and tramadol), and extrapolated prevalence to estimated affected US population.

**Results:**

The NHANES contained 20,146 participants, of whom 135 reported taking ≥1 antiseizure medication (ASM) for seizures or epilepsy representing 2,399,520 US citizens using NHANES's sampling frame. Patients reported taking a mean 5.3 (95% confidence interval (CI): 4.3–6.3) prescription medications. Adjusting for race, sex, and uninsurance, both age and number of chronic conditions predicted increased number of medications (incident rate ratio (IRR) per decade: 1.16, 95% CI: 1.04–1.28; IRR per chronic condition: 1.19, 95% CI: 1.11–1.27). Polypharmacy was reported by 47% (95% CI: 38%–57%) of patients, CNS polypharmacy by 34% (23%–47%), benzodiazepine use by 21% (14%–30%), opioid use by 16% (11%–24%), benzodiazepine plus opioid use by 6% (3%–14%), and 6% (2%–15%) reported a drug that lowers the seizure threshold. Twelve percent (7%–20%) took an opioid with either a benzodiazepine or gabapentinoid.

**Conclusions:**

Polypharmacy is common in patients with epilepsy. Patients taking ASMs frequently reported also taking other CNS-acting medications (i.e., opioids, benzodiazepines, seizure threshold-lowering medications), and medication combinations with black box warnings. Central nervous system polypharmacy poses health risks. Future research is needed to explore drivers of polypharmacy and strategies to help mitigate potentially harmful prescription use in this high-risk population.

## 1. Introduction

Patients with epilepsy experience a high degree of comorbidity [1], and comorbidities drive polypharmacy [2]. While multiple prescriptions may be appropriate for patients with many chronic conditions, more prescriptions also create the potential for inappropriate prescribing [3], which is associated with adverse outcomes such as poor health, hospitalization, and death [4-9]. Patients with epilepsy are known to have increased risk for medication self-poisoning especially related to opioids and psychotropic medications [10]. Moreover, one study found among patients with epilepsy that 25% of variance in quality of life was explained simply by medication adverse effects [11].

While patients with epilepsy likely experience a high burden of polypharmacy, the extent of the problem is poorly understood. Only limited data exist describing the frequency and composition of polypharmacy and central nervous system (CNS)-acting medications taken by patients with epilepsy beyond simply antiseizure medications (ASMs) [12-15]. However, certain knowledge gaps exist. No nationally representative data exist in the US regarding polypharmacy in patients with epilepsy. Furthermore, these prior studies did not explicitly capture particularly important medication combinations such as seizure threshold-lowering medications or certain ‘black box warnings’ including combinations of opioids and benzodiazepines or gabapentinoids. Finally, such studies more narrowly defined ‘polypharmacy’ or ‘concomitant medications’ as >1 ASM or 1 ASM plus ≥1 non-ASM, whereas general medical literature more typically defines polypharmacy in broader terms of total number of medications exceeding a certain threshold such as ≥5 medications [16,17]. Thus, we currently lack a wider-view examination of total number of prescription medications and relevant potentially dangerous combinations taken by people with epilepsy especially in the US. Further characterizing the regimens currently prescribed to patients with epilepsy including but also beyond ASMs is a critical step towards future investigation aimed at identifying potentially inappropriate drug treatments and reducing adverse effects from complex CNS polypharmacy.

In this study, we used nationally representative US survey data to describe the number and types of prescription medications used by patients with epilepsy. First, we described the total number of medications and examined several a priori specified key predictors. Then, we studied the frequency of polypharmacy overall. Finally, given unique risks of CNS-active medications and particular drug–drug combinations, we examined the frequency of CNS polypharmacy, opioids, benzodiazepines, gabapentinoids, medications known to lower the seizure threshold, and several combinations with known hazards.

## 2. Methods

### 2.1. Study design and dataset

This was a cross-sectional analysis of the National Health and Nutrition Examination Survey (NHANES) using data collected from 2013 to 2016 during which participants were asked for the indications of each prescription medication. The NHANES is a long-standing semiannual cross-sectional study run by the Centers for Disease Control and Prevention. Its goal is to understand broad trends in health and nutrition in the United States. The NHANES samples approximately 5000–10,000 noninstitutionalized individuals from 15 counties across the US each year and oversamples certain individuals (over 60 years old, African Americans, Hispanics) selected from the US Census to ensure it is nationally representative. It uses complex, stratified, multistage probability cluster sampling and collects data including respondents' prescribed medications and health conditions. Health interviews are conducted in a participant's home, and in-person physical examination by a physician is conducted in a traveling mobile center. The design and operation of NHANES are available online (https://wwwn.cdc.gov/nchs/nhanes/default.aspx).

### 2.2. Procedures involving human subjects

This study was deemed exempt by the University of Michigan Institutional Review Board, given use of publicly available deidentified datasets.

### 2.3. Patient selection

The NHANES collects information about all participants' prescription medications. Each participant listed the name of each medication they have taken in the last 30 days prescribed by a health professional. Participants provided up to 3 main reasons for using each medication.

We limited analysis to survey participants who responded that they were taking at least one medication for “epilepsy and recurrent seizures” (G40). We confirmed that each medication was a standard ASM. If a medication was coded as G40 but not actually an ASM by manual review, we converted the International Classification of Diseases (ICD) indication to blank and did not count such medications towards our case definition.

### 2.4. Variables

We collected baseline variables related to polypharmacy in order to describe our population [2]. Demographics included age, sex, and race. Income-to-poverty ratio represents a family's income as a ratio of poverty guidelines. Participants reported whether a healthcare professional had diagnosed a variety of conditions including asthma, chronic obstructive pulmonary disease, congestive heart failure, coronary disease, hypertension, diabetes, liver disease, thyroid disease, and malignancy. Epilepsy was not specifically asked about in this section. For the definition of hypertension and diabetes, we required that participants either reported at least one medication treating these conditions, or else NHANES measurements suggested the diagnosis (hypertension: systolic blood pressure (SBP) > 140 or diastolic blood pressure (DBP) > 90 averaged over 3 measures; diabetes: A1c > 7%) as has been done in prior NHANES studies [18]. Patients also completed a Patient Health Questionnaire-9 (PHQ9) to evaluate depression severity at the time of the survey. Other variables included insurance coverage, household income, and self-reported health status.

### 2.5. Statistical analysis

For categorical data, we report raw counts and survey-weighted proportions and 95% confidence intervals (CIs). For continuous data, we report survey-weighted means plus standard deviation (SD) or 95% CIs. The weights provided in each biennial cycle's dataset were divided by 2 (the number of interview cycles we have used: 2013–2014 and 2015–2016), so that the estimates are nationally representative of the US population across the four-year time period [19].

For our main analysis, we counted each patient's total number of reported medications. We report the most common medication names, therapeutic category and primary disease systems using Multum Lexicon®, and patient-reported indications. We then performed several analyses identifying predictors of total medication count. First, we displayed scatterplots of total number medications according to two prespecified predictors identified based on literature [2] and theoretical importance — age, and total number of chronic conditions. Second, we performed an adjusted regression to assess the association between these variables and total number of medications. To accomplish this, we conducted a survey-weighted zero-truncated negative binominal regression. In this model, the total number of medications was the outcome variable, and predictors included age and number of chronic conditions, adjusted also for race, uninsurance, and sex. The chosen model was a negative binomial regression given medication data were overdispersed and zero-truncated given all participants entering this study by definition reported at least 1 medication. After analyzing medication count as a continuous variable, we classified this number as polypharmacy (5 or more) and also at least 10 medications [16,17].

We further explored a priori particular types and combinations of medications. We applied the Multum Lexicon® to define opioids and benzodiazepines given their special importance in polypharmacy. We flagged whether benzodiazepines were prescribed for epilepsy, versus a nonseizure indication. We defined CNS polypharmacy as at least 3 CNS-acting medications, according to updated Beers criteria [20,21]. Antiseizure medications were counted towards this definition (thus, all patients in this study were on at least 1 CNS-acting medication), in addition to antipsychotics, benzodiazepines, nonbenzodiazepine benzodiazepine receptor agonists, tricyclic antidepressants, selective serotonin reuptake inhibitors (SSRIs), selective serotonin–norepinephrine reuptake inhibitors (SNRIs), or opioids. We lastly evaluated particular disease–drug interactions (including common medications known to lower the seizure threshold: tramadol [22,23] and bupropion [24,25]) plus several drug–drug combinations with black box warnings from the US Food and Drug Administration (opioid-benzodiazepine [26]; opioid-gabapentinoid [27]). We calculated the survey-weighted percentage of our sample taking each of the above medications or combinations and multiplied this percentage times the total US population represented by our included participants to estimates the involved US population accounting for NHANES's complex survey weighting design.

Data were analyzed using SAS 9.4 (Cary, NC) and Stata 14.2 (College Station, TX).

### 2.6. Data accessibility statement

All datasets are freely available for download at https://wwwn.cdc.gov/nchs/nhanes/Default.aspx.

## 3. Results

Combining the 2013–2014 and 2015–2016 NHANES samples, there were 20,146 participants. Of these, 136 (0.7%) had at least one medication reported for “epilepsy and recurrent seizures”. Three participants reported medications for “epilepsy and recurrent seizures”, which were not actually an ASM: hydrocodone, allopurinol, and apixaban. Two of these 3 cases were still included because they endorsed at least 1 other true ASM for “epilepsy and recurrent seizures.” The remaining case was excluded because there was no true ASM for “epilepsy and recurrent seizures.” Thus, our final sample size was 135 (raw proportion: 135/20,146 = 0.7%; survey-weighted proportion: 0.8%, 95% CI: 0.6%–0.9%) representing 2,399,520 US citizens using NHANES's sampling frame.

The mean age was 51 (SD: 15), 58% were male, 70% were non-Hispanic white, and 8% were uninsured. The mean number of nonepilepsy comorbidities of those listed available in NHANES was 3.8 (SD: 2.8) (Table 1). Of the 135 participants, 70% (95% CI: 55%–79%) reported one ASM, 21% (14%–29%) two ASMs, 6% (2%–19%) three ASMs, and 3% (1%–15%) four ASMs. The most common medications, indications, and medication classes are shown in Supplemental Tables 1, 2, and 3, respectively.

**Fig. 1.**
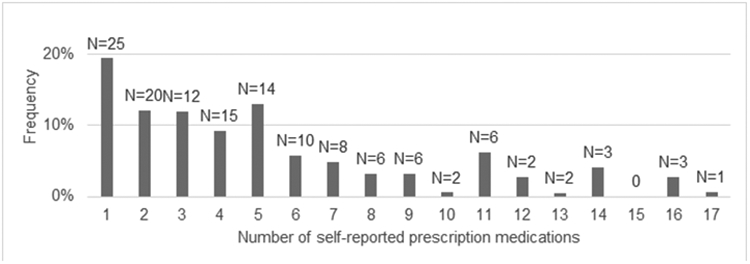
Distribution of total medications. Frequencies (%) are weighted. Counts (N) above each bar are raw.

**Fig. 2.**
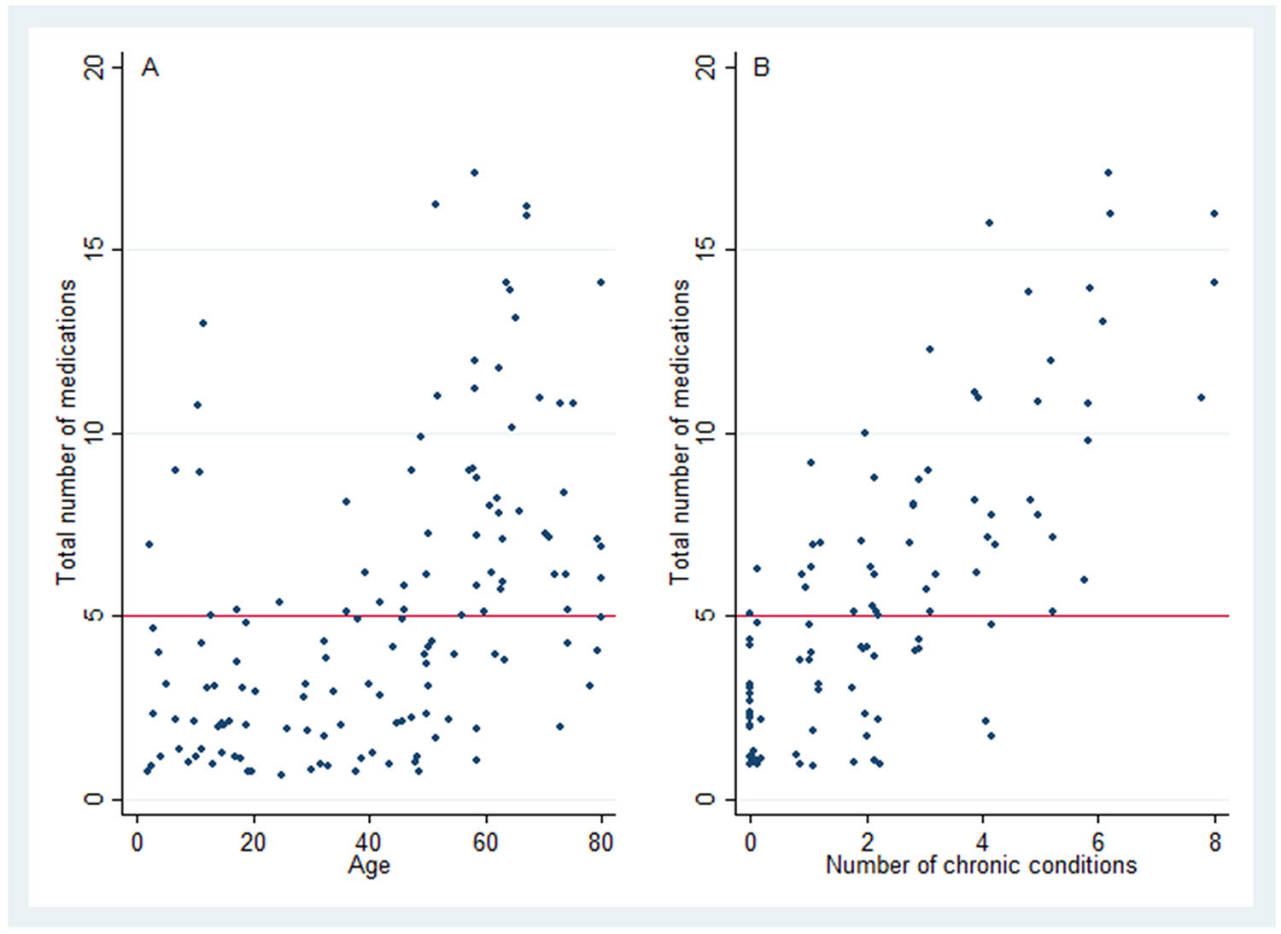
Bivariate relationship between total medications and A) age (N=135) and B) number of chronic conditions (N=99, given chronic conditions were only obtained from participants ≥20 years old). The horizontal line at Y=5 denotes the cutoff at/above which participants are classified as having polypharmacy. Note there is a small amount of jitter for display given overlapping data points (i.e., number of chronic conditions take on integer values). Number of chronic conditions was calculated as the sum of the following conditions: chronic obstructive pulmonary disease, congestive heart failure, coronary disease, hypertension, diabetes, liver disease, thyroid disease, and malignancy. These conditions were self-reported, except for hypertension and diabetes whose definitions also included blood pressure or A1c measurement, or else pharmacotherapy as reported in the Methods section.

**Fig. 3.**
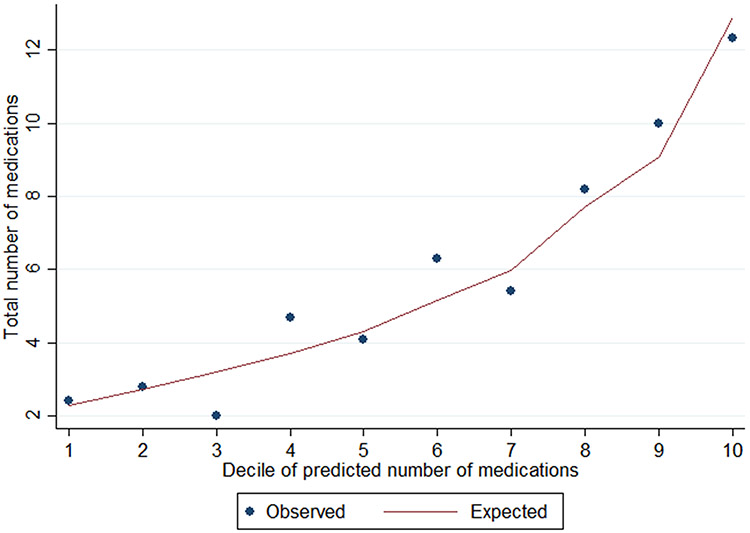
Model calibration. For the survey-weighted zero-truncated negative binomial regression, we modeled how total medications was predicted by age, number of chronic conditions, race, uninsurance, and sex (N = 99). To assess model fit, we calculated predicted total medication count from the model, and in this figure plot, the observed and expected medication count for each decile of predicted medication count. This figure shows the model demonstrates good calibration across the population.

The mean number of total prescription medications was 5.3 (95% CI: 4.3–6.3). Fig. 1 depicts the distribution of total medications in our sample. Excluding ASMs, the mean (95% CI) number of medications was 3.8 (2.8–4.8). Fig. 2 contains scatterplots depicting the bivariate relationship between total prescription medications and the following two variables: age and number of nonepilepsy chronic conditions. Table 2 displays results from a zero-truncated negative binomial regression including each of the following predictors: age, number of nonepilepsy chronic conditions, race, uninsurance, and sex. For every decade, there was a 16% increase in number of medications (incident rate ratio (IRR): 1.16; 95% CI: 1.04–1.28). For every additional chronic condition, there was a 19% increase (IRR: 1.19; 95% CI: 1.11–1.27). Fig. 3 shows good model calibration between observed and expected values across deciles of predicted medication count.

Table 3 demonstrates prevalence of polypharmacy (≥5 medications) as a dichotomous variable and our a priori specific medications and medication combinations of interest. Forty-seven percent (95% CI: 38%–57%) of participants met criteria for polypharmacy and 17% (9%–32%) for at least 10 medications. Using NHANES's sampling frame, these frequencies extrapolate to 1,137,612 US citizens treated for epilepsy taking 5 or more medications, which includes 419,676 taking 10 or more medications. Twenty-one percent (95% CI: 14%–30%) of participants reported at least one benzodiazepine, 16% (11%–24%) at least one opioid, and 6% (2%–15%) at least one medication known to lower the seizure threshold (bupropion 2%; tramadol 4%). Regarding specific drug combinations, 6% (3%–14%) reported an opioid plus benzodiazepine, 7% (4%–13%) an opioid plus gabapentinoid, and 34% (23%–47%) CNS polypharmacy. Table 3 also displays in absolute terms how many US citizens each of these percentages represent; for example, this represents 502,219 US citizens using a benzodiazepine, 386,802 using an opioid, and 819,436 fulfilling criteria for CNS polypharmacy.

## 4. Discussion

In a nationally representative sample, we found that patients treated for epilepsy took an average 5.3 medications, which increased with age and chronic condition burden. Approximately half met criteria for polypharmacy, 17% reported at least 10 medications, and 34% had CNS polypharmacy. We documented that 21% reported at least 1 benzodiazepine, 16% reported at least one opioid, 12% reported an opioid in combination with either a benzodiazepine or gabapentinoid despite the combinations' known danger, and 6% reported at least one medication known to lower the seizure threshold. Extrapolating to the US population using NHANES's sampling frame, these frequencies represent approximately 1.1 million US citizens treated for epilepsy taking 5 or more medications. This estimate includes over 500,000 using at least 1 benzodiazepine, 387,000 using at least 1 opioid, 248,000 using a black box warning opioid combination, 150,000 taking medications known to lower the seizure threshold (tramadol, bupropion), and 819,000 using at least 3 CNS-acting medications. These results suggest that sizable numbers of patients with epilepsy are potentially at risk for adverse effects from polypharmacy.

These findings are concerning. Prior work has shown that patients with epilepsy demonstrate 3–5 times increased medication self-poisoning compared with populations without epilepsy [10]. In that study, opioids and psychotropic medications were more commonly involved in poisoning-related deaths than ASMs, which underscores the importance of monitoring pharmacoepidemiology more broadly than just ASMs in patients with epilepsy. In line with this observation, over 70,000 deaths occur each year in the US due to drug overdose, and two-thirds of these are related to opioids [28]. A prior study using all NHANES participants [29] estimated 7% of US adults took an opioid. The 16% (95% CI: 11%–24%) prevalence of opioids in our study suggests that opioid use is increased in patients with epilepsy. In a privately insured population, prevalence of opioid use was 26% with epilepsy versus 18% without epilepsy [12]. Opioids were the second most common class of medications among patients with epilepsy after ASMs [13]. In their study, consistent with prior work [30], patients with epilepsy did demonstrate increased pain and psychiatric conditions that could drive this relationship. Our study expands upon this prior work by utilizing a nationally representative dataset that includes uninsured patients, explores important drug combinations, and captures drugs that lower the seizure threshold and particularly CNS polypharmacy. Given the magnitude of the problem, future attention needs to focus on mechanisms driving high opioid usage among patients with epilepsy and the development of alternative strategies for analgesia.

Benzodiazepine use is likewise common and potentially harmful because of potential dependence, somnolence, and respiratory suppression [31]. Approximately 5–10% of US adults are prescribed a benzodiazepine [32,33]. Increased anxiety and depression in patients with epilepsy [34] could explain increased benzodiazepine use. We did find that most benzodiazepines in this study were used to treat seizures. While benzodiazepines are indicated for acute seizure treatment and can be effective for seizure reduction, our study nonetheless highlights that prescribers must remain cognizant of a patient's overall medication regimen in order to avoid potentially harmful interactions. Additionally, we found 12% of participants combined opioids with a gabapentinoid or benzodiazepine, despite a current black box warning. For example, opioids plus benzodiazepines pose well-known risk: 23% of opioid-related overdoses also involve benzodiazepines, concurrent use elevates risk for emergency room (ER) use and hospitalization [35], and overdose death rates are 10 times higher for those coprescribed opioids and benzodiazepines compared with those prescribed opioids alone [36].

This study highlights the broader issue of CNS polypharmacy. An estimated 4 million outpatient visits with CNS polypharmacy occur each year for patients 65 years and older, which has been rising over time [21], and literature has documented cumulative toxicity and drug interactions due to CNS polypharmacy [37,38]. Prior work has provided important background information; for example, one large study in Norway found 37% of patients on an ASM used at least 1 other ASM, antidepressant, or antipsychotic [15]. Our study builds upon this prior work, and other work examining prevalence of concomitant medications taken alongside ASMs [14], because our study captured polypharmacy as more broadly defined in the medical literature, and identified particular dangerous medication combinations and drug–disease interactions that were not described in prior studies. Of course, many patients have legitimate indications for multiple CNS-acting medications, and in each situation, the neuropsychiatric benefits must be weighed against additive risk. However, exploring CNS polypharmacy as we have done here is particularly useful to shed light on a potentially modifiable source of adverse outcomes.

Finally, it is worth noting that ASMs comprised the largest group of medication in this study. We did define epilepsy according to ASM use, so this result is expected. Nonetheless, ASMs are the mainstay of treatment for epilepsy and contribute substantially to CNS polypharmacy. Adverse effects of ASMs range from 10 to 40% of patients in unstructured screening to as high as 60–90% [39] in structured screenings, and adverse effects are correlated with worsened quality of life [40-43]. This underscores the need to carefully consider if and when ASMs are no longer necessary. Two-thirds of patients with epilepsy are well-controlled on medications [44] and may be candidates for ASM withdrawal [45], and ASM discontinuation for seizure-free patients has been associated with improvement in key outcomes such as mood [46], cognition [47-49], and psychosocial well-being [50]. Accordingly, ASM treatment decisions represent a ready lever under the clinician's control.

This study has several limitations. Inclusion criteria were based on self-reported treatment indications, which could misclassify patients. For example, our inclusion criteria based on available data (taking ≥1 ASM for seizures or epilepsy) may not have captured all patients with epilepsy; patients with well-controlled epilepsy may eventually appropriately discontinue their ASM [45] or else patients may inappropriately not receive an ASM despite it being indicated (the “treatment gap”) [51]. However, we believe that this is a reasonable definition because identification of epilepsy by self-report has been validated with positive predictive value of 74% and sensitivity of 84% [52], and the presence of an ASM has been shown to substantially improve detection of epilepsy in research datasets [53], data exist validating accuracy of self-reported analgesia use [54], and ASMs are the mainstay of treatment for epilepsy. Self-report actually presents a distinct advantage: whereas chart listing or prescription claims suggest a patient has been prescribed a medication, self-report reflects what a patient has actually been taking. Second, our data do not distinguish between intermittent rescue versus daily chronic use of benzodiazepine or opioids. Third, our sample size of 135 participants leads to certain estimates having wide confidence intervals, for example, seizure threshold-lowering drugs. Nonetheless, these 135 participants actually extrapolate to 2.4 million US individuals given NHANES's complex, careful sampling design, which is a major strength.

## 5. Conclusions

Medication burden is high in patients with epilepsy. This includes overall polypharmacy, CNS polypharmacy, and both potential drug–drug and drug-disease interactions. Future work is needed to clarify the drivers of polypharmacy in epilepsy, further understand downstream effects of polypharmacy in this group, and develop interventions to reduce potentially inappropriate or harmful medication burden in this high-risk population.

## Supplementary Material

supplementary tables

## Figures and Tables

**Table 1:** Population description. N = 135.

		Mean (SD) or No. (%)^a^
Demographics	Age	51 (15)
	Male sex	75 (58%)
	Race	
	Mexican American	17 (8%)
	Non-Hispanic black	21 (8%)
	Non-Hispanic white	62 (70%)
	Uninsured	12 (8%)
	Family income to poverty ratio^b^	1.7 (1.4)
Comorbidities^c^	Asthma	33 (26%)
	Cancer	18 (16%)
	Chronic obstructive pulmonary disease	21 (23%)
	Congestive heart failure	7 (14%)
	Coronary disease	6 (11%)
	Diabetes mellitus	17 (14%)
	Hypertension	51 (38%)
	Liver disease	7 (11%)
	Patient Health Questionnaire 9 (PHQ9)^d^	5.6 (5.7)
	Stroke	21 (19%)
	Thyroid disease	17 (20%)
	Number chronic conditions	3.8 (2.8)

a Frequencies are raw counts. All means (SD) and percentages are weighted according to the NHANES sampling frame. Variables have 0% missingness for those raw N= 135 in the sample except as stated below.

b Income had weighted 6% missing data (raw included N/raw eligible N = 125/135).

c Comorbidities were only asked of patients at least 20 years old, with 0% missing (raw included N/raw eligible N= 99/99).

d PHQ9 was asked of those at least 18 years old, with weighted 19% missing data (raw included N/raw eligible N = 81/104). Common interpretation thresholds for depression include 0–4minimal, 5–9mild, 10–14moderate, 15–19moderately severe, and 20–27 severe [55].

**Table 2:** Predictors of total number of medications. N = 99. The following displays results from a survey-weighted zero-truncated negative binomial regression. This model estimates the incident rate ratio for each predictor adjusted for all other listed predictors, on total number of medications. Estimates may be interpreted as the relative increase in total number of medications for each 1-unit change in the listed predictor, adjusted for all others. Note that only participants ≥20 years old (N=99) provided responses to listed chronic conditions, out of our total study population (N= 135).

		IRR % (95% CI)^a^
Age, decade		1.16 (1.04–1.28)*
Number of chronic conditions^b^		1.19 (1.11–1.27)*
Race	Non-Hispanic white	Ref
	Non-Hispanic Black	0.90 (0.63–1.30)
	Mexican American	1.11 (0.75–1.66)
	Other Hispanic	0.76 (0.46–1.23)
Uninsured		1.03 (0.79–1.37)
Male		1.11 (0.83–1.49)

a All estimates are adjusted for each of the other listed variables.

b Number of chronic conditions was calculated as the sum of the following conditions: chronic obstructive pulmonary disease, congestive heart failure, coronary disease, hypertension, diabetes, liver disease, thyroid disease, and malignancy. These conditions were self-reported, except for hypertension and diabetes whose definitions also included blood pressure or A1c measurement, or else pharmacotherapy as reported in the methods section.

* p < 0.05.

**Table 3:** Specific medications.

		Raw no.	Weighted % (95% CI)	Weighted no.^a^
Overall	≥5 medications (polypharmacy)	63	47% (38%–57%)	1,137,612
	≥10 medications	19	17% (9%–32%)	419,676
Specific medications	≥1 benzodiazepine	30	21% (14%–30%)	502,219
	≥1 benzodiazepine, not for epilepsy or seizures	11	7% (3%–14%)	162,207
	≥1 opioid	23	16% (11%–24%)	386,802
	Bupropion	1	2% (<1%–14%)	50,629
	Tramadol	8	4% (2%−11%)	100,539
Medication combinations	Opioid plus benzodiazepine	8	6% (3%–14%)	147,330
	Opioid plus gabapentinoid	7	7% (4%–13%)	166,047
	Opioid plus either benzodiazepine or gabapentinoid	14	12% (7%–20%)	248,830
	CNS polypharmacy^b^	47	34% (23%–47%)	819,436

a Weighted number refers to the weighted percentage (% from the middle column) times the weighted sample (N=2,399,520). This is because the 135 included partidpants represent 2,399,520 US citizens per NHANES's sampling frame.

b CNS polypharmacy: At least 3 CNS-acting medications including antiepileptics, antipsychotic, benzodiazepine, nonbenzodiazepine benzodiazepine receptor agonist, tricyclic antidepressant, selective serotonin reuptake inhibitor, selective serotonin-norepinephrine reuptake inhibitor, and opioids, as defined in Beers criteria [20].
