# Supplementary material for: Polypharmacy in patients with epilepsy: A nationally representative cross-sectional study
Source: Epilepsy Behav. Author manuscript; Available in PMC 2021 Feb 8. (PMC7869064; doi:10.1016/j.yebeh.2020.107261)
Supplement: supplementary tables [file NIHMS1664781-supplement-supplementary_tables.docx]

**Supplemental Table 1:** Most common medications, descending weighted percentage

|  | **Raw No.** | **Weighted % (95% CI)** | **Weighted No.^a^** |
| --- | --- | --- | --- |
| Lamotrigine | 24 | 27% (17%-40%) | 642,352 |
| Levetiracetam | 38 | 23% (14%-37%) | 562,927 |
| Phenytoin | 27 | 19% (12%-30%) | 463,347 |
| Gabapentin | 17 | 17% (12%-25%) | 413,677 |
| Divalproex | 19 | 17% (10%-28%) | 404,319 |
| Metoprolol | 16 | 15% (9%-22%) | 350,090 |
| Lisinopril | 14 | 14% (8%-22%) | 328,014 |
| Carbamazepine | 12 | 13% (6%-27%) | 307,618 |
| Omeprazole | 14 | 11% (4%-23%) | 252,909 |
| Amlodipine | 14 | 10% (5%-22%) | 250,030 |
| Levothyroxine | 9 | 10% (4%-22%) | 242,352 |
| Atorvastatin | 12 | 10% (4%-21%) | 230,834 |
| Topiramate | 13 | 8% (6%-13%) | 201,080 |
| Metformin | 14 | 8% (5%-13%) | 195,801 |
| Simvastatin | 8 | 7% (2%-19%) | 158,608 |
| Hydrochlorothiazide | 10 | 6% (3%-12%) | 143,491 |
| Lacosamide | 8 | 5% (2%-13%) | 120,216 |
| Diazepam | 9 | 4% (2%-10%) | 103,659 |
| Phenobarbital | 8 | 4% (1%-9%) | 85,183 |
| Oxcarbazepine | 8 | 2% (1%-5%) | 51,590 |

^a^Weighted number refers to the weighted percentage (% from the middle column) times the weighted sample (N= 2,399,520). This is because the 135 included participants represent 2,399,520 US citizens per NHANES’s sampling frame.

**Supplemental Table 2:** Most common indications for prescription medications

|  | **Raw No.** | **Weighted % (95% CI)** | **Weighted No.^a^** |
| --- | --- | --- | --- |
| Epilepsy and recurrent seizures | 135 | 100% (N/A) | 2,399,520 |
| Hypertension | 46 | 34% (24%-45%) | 810,558 |
| Pure hypercholesterolemia | 29 | 23% (16%-32%) | 554,289 |
| Major depressive disorder | 19 | 15% (8%-28%) | 368,086 |
| Dorsalgia | 13 | 15% (9%-23%) | 360,888 |
| Gastro-esophageal reflux disease | 21 | 13% (8%-22%) | 321,296 |
| Type 2 diabetes mellitus | 16 | 13% (7%-25%) | 320,096 |
| Anxiety disorder | 15 | 13% (6%-24%) | 300,900 |
| Neuralgia and neuritis | 10 | 10% (5%-17%) | 232,753 |
| Insomnia | 12 | 9% (3%-22%) | 210,678 |
| Hypothyroidism | 7 | 7% (3%-15%) | 159,568 |
| Sleep disorder | 9 | 5% (2%-11%) | 115,657 |
| Allergic rhinitis | 8 | 4% (1%-14%) | 102,460 |
| Migraine | 6 | 4% (1%-15%) | 102,220 |
| Myalgia | 6 | 4% (1%-12%) | 91,902 |
| Allergy | 8 | 4% (2%-7%) | 89,022 |
| Constipation | 7 | 4% (1%-11%) | 87,582 |
| Asthma | 8 | 3% (2%-7%) | 81,344 |
| Wheezing | 3 | 1% (0%-5%) | 29,514 |

^a^Similar to Supplemental Table 1.

**Supplemental Table 3:** Most common primary drug categories

|  | **Raw No.** | **Weighted % (95% CI)** | **Weighted No.^a^** |
| --- | --- | --- | --- |
| Central nervous system | 127 | 94% (86%-97%)^b^ | 2,253,629 |
| Psychotherapeutic | 45 | 65% (54%-74%) | 1,561,368 |
| Cardiovascular | 67 | 50% (38%-62%) | 1,197,840 |
| Metabolic | 38 | 30% (21%-40%) | 709,778 |
| Gastrointestinal | 32 | 23% (14%-34%) | 540,612 |
| Hormonal | 17 | 15% (8%-28%) | 370,006 |
| Respiratory | 20 | 13% (7%-23%) | 315,297 |
| Anti-infective | 12 | 10% (5%-17%) | 231,794 |
| Coagulation | 14 | 9% (4%-21%) | 220,516 |
| Nutritional | 5 | 7% (3%-13%) | 165,567 |
| Topical | 12 | 6% (2%-14%) | 140,132 |
| Miscellaneous | 5 | 4% (1%-13%) | 99,820 |
| Antineoplastic | 3 | 3% (1%-8%) | 62,627 |
| Genitourinary | 1 | 1% (0%-5%) | 17,037 |
| Alternative medications | 1 | 0% (0%-2%) | 5,519 |

^a^Similar to Supplement Tables 1 and 2.

^b^Note that Multum Lexicon® classifies phenytoin primarily as a cardiovascular agent as an antiarrhythmic, thus the first row is not 100%.
